# Multimodal Finger Pulse Wave Sensing: Comparison of Forcecardiography and Photoplethysmography Sensors

**DOI:** 10.3390/s22197566

**Published:** 2022-10-06

**Authors:** Emilio Andreozzi, Riccardo Sabbadini, Jessica Centracchio, Paolo Bifulco, Andrea Irace, Giovanni Breglio, Michele Riccio

**Affiliations:** Department of Electrical Engineering and Information Technologies, University of Naples Federico II, Via Claudio, 21, 80125 Napoli, Italy

**Keywords:** arterial pulse wave, finger pulse, photoplethysmography, forcecardiography, piezoelectric sensor, multimodal sensor, pulse oximetry

## Abstract

Pulse waves (PWs) are mechanical waves that propagate from the ventricles through the whole vascular system as brisk enlargements of the blood vessels’ lumens, caused by sudden increases in local blood pressure. Photoplethysmography (PPG) is one of the most widespread techniques employed for PW sensing due to its ability to measure blood oxygen saturation. Other sensors and techniques have been proposed to record PWs, and include applanation tonometers, piezoelectric sensors, force sensors of different kinds, and accelerometers. The performances of these sensors have been analyzed individually, and their results have been found not to be in good agreement (e.g., in terms of PW morphology and the physiological parameters extracted). Such a comparison has led to a deeper comprehension of their strengths and weaknesses, and ultimately, to the consideration that a multimodal approach accomplished via sensor fusion would lead to a more robust, reliable, and potentially more informative methodology for PW monitoring. However, apart from various multichannel and multi-site systems proposed in the literature, no true multimodal sensors for PW recording have been proposed yet that acquire PW signals simultaneously from the same measurement site. In this study, a true multimodal PW sensor is presented, which was obtained by integrating a piezoelectric forcecardiography (FCG) sensor and a PPG sensor, thus enabling simultaneous mechanical–optical measurements of PWs from the same site on the body. The novel sensor performance was assessed by measuring the finger PWs of five healthy subjects at rest. The preliminary results of this study showed, for the first time, that a delay exists between the PWs recorded simultaneously by the PPG and FCG sensors. Despite such a delay, the pulse waveforms acquired by the PPG and FCG sensors, along with their first and second derivatives, had very high normalized cross-correlation indices in excess of 0.98. Six well-established morphological parameters of the PWs were compared via linear regression, correlation, and Bland–Altman analyses, which showed that some of these parameters were not in good agreement for all subjects. The preliminary results of this proof-of-concept study must be confirmed in a much larger cohort of subjects. Further investigation is also necessary to shed light on the physical origin of the observed delay between optical and mechanical PW signals. This research paves the way for the development of true multimodal, wearable, integrated sensors and for potential sensor fusion approaches to improve the performance of PW monitoring at various body sites.

## 1. Introduction

Pulse waves (PW) or sphygmic waves are mechanical waves that propagate from the ventricles through the whole vascular system as brisk enlargements of the blood vessels’ lumens, caused by sudden increases in local blood pressure [1,2,3]. Photoplethysmography (PPG) is one of the most widely used techniques for PW sensing, owing to its ability to measure blood oxygen saturation when both red and infrared light sources are used simultaneously. PPG is commonly used both in clinical and non-clinical settings, and it has been integrated in a variety of consumer electronics products to enable wearable, unobtrusive monitoring of heart rate and arrhythmias [1,2,3].

PPG monitors blood volume variations in a microvascular bed of the skin non-invasively, by measuring the related changes in the optical absorption, scattering, and transmission properties of human tissues under a specific light wavelength [4]. In particular, PPG records the amount of light transmitted or reflected by tissues, which varies over time according to pulsation, as it causes changes in the optical path and in the amount of light absorbed by the monitored body part. To this end, PPG sensors irradiate light at specific wavelengths into the skin, which passes through various skin structures (tissues, veins, arteries) and is finally measured by a photodetector [1,2,3]. The measured light intensity depends upon several factors, such as the blood volume at the measurement site, skin pigmentation and composition, the arterial diameter, and the concentration and direction of oxygenated and deoxygenated hemoglobin [5,6,7]. Light sources with various wavelengths can be used, with the depth of penetration usually increasing with the wavelength [8,9]. As an example, by increasing the wavelength from 470 nm (which allows it to reach the epidermis with capillaries) to 570 or 660 nm, the irradiated light can penetrate to the dermis with arterioles, and down to arteries in subcutaneous tissues, respectively [10,11]. As major blood vessels with solid pulsations are mainly located in the dermis or subcutaneous tissues, light sources with red (640–660 nm) and infrared (880–940 nm) wavelengths are commonly used in PPG sensors [1,12].

PPG sensors usually feature one or more light-emitting diodes (LED) and wavelength-matched photodetectors. According to the positioning of light sources and detectors on the monitored body part, PPG sensors can be divided into the transmissive type and reflective type. Transmissive PPG sensors have the source and detector placed at opposite sides of the monitored body part and measure the intensity of the transmitted light, while reflective PPG sensors have the source and detector placed next to each other on the same side of the monitored body part and measure the intensity of the reflected light [1]. Reflective PPG sensors show less stable performance than transmissive ones [1,13]. On the other hand, reflective PPG sensors are not restricted to distal, thinner body parts, but can also monitor blood volume changes in other body districts where light transmission is difficult [1]. Indeed, PPG is usually acquired for peripheral body districts, especially extremities such as the fingers, toes, and earlobes, because their shallow vascular beds ensure high-quality measurements of blood volume changes [14,15,16]. This is particularly true for transmissive PPG sensors, as body districts with small widths ensure reasonable transmitted light intensities, while thicker body districts absorb too much light, thus resulting in unsuitable signal-to-noise ratios. However, reflective PPG sensors can also be applied on the forehead, face, nose, and esophagus [17,18,19,20], as well as on shallow arteries of thicker body parts, such as carotid arteries in the neck, radial and ulnar arteries in the wrist, brachial arteries in the arms, and femoral arteries in the thighs [21,22,23].

The low cost and unobtrusiveness of PPG sensors has promoted their use in a broad spectrum of applications, the most popular ones undoubtedly being pulse oximetry, i.e., measurement of saturation of peripheral oxygen (SpO_2_) with the combined use of red and infrared wavelengths and heart rate monitoring [1,2,3]. Other clinical applications of PPG sensors include: blood pressure estimation [24,25,26,27,28,29,30,31,32,33]; the assessment of vascular aging [34,35,36,37,38,39] and peripheral vascular disease [40,41,42,43]; the monitoring of respiratory-induced changes in peripheral blood flow and the correlation between intrathoracic pressure and cardiac function in patients with heart failure or respiratory distress [44,45]; sleep monitoring for the detection of apnea and hypopnea conditions [46,47,48,49]; the estimation of stroke volume and cardiac output [50,51,52,53]; and the detection of atrial fibrillation [54].

The typical pulse waveform recorded via PPG sensors is obtained by inverting the light intensity signal acquired by the photodetector, and is commonly divided into pulsatile and non-pulsatile components [1,3,55]. The pulsatile component is correlated with the cardiac cycle and influenced by vasodilation, vasomotor, and vascular tones, as well as respiration and autonomic nervous system activity [56,57,58,59,60,61,62,63,64]. The non-pulsatile component is influenced by ambient light [65], biological characteristics such as tissue composition and basic blood volume of the measurement site, and various physiological phenomena such as respiration, vasomotor activity, and thermoregulation [66,67,68,69]. Skin color, blood vessel distribution, vascular stiffness, oxygen-carrying capacity, bone size, and cardiac output are also known to affect the amplitude of PPG signals [70,71]. For these reasons, the analysis of PPG waveforms is still an important subject of scientific research. In addition, the physiological relevance of first and second derivatives of PPG signals (also known as velocity and acceleration PPG, respectively) has been recognized since the 1970s [1]. As an example, it has been shown that indices extracted from peaks and valleys of the second derivative of a PPG signal have significant correlations with aging [39]. In addition, PPG derivative signals support the robust recognition of specific fiducial markers (e.g., peaks and valleys, inflection points, and points of maximal slope), which can be difficult to locate in the original PPG waveform [1].

Applanation tonometry (AT) is another well-established technique for PW recording [72]; however, it is not suitable for wearable applications, being based on a hand-held device. AT is usually adopted in PW velocity (PWV) measurements (i.e., the measure of PW propagation velocity along a blood vessel), which is a surrogate measurement of arterial stiffness and provides important information about cardiovascular disease outcomes [73]. Tonometry can also be used to measure the blood pressure waveform in superficial arteries, which must be maintained in an applanated state over time via a controlled force. However, this condition is very difficult to obtain in practice, and calls for frequent calibrations [74].

Unlike applanation tonometry, piezoelectric sensors capture PW signals without the need for arteries to be flattened, thus being suitable for wearable, long-term monitoring of PWs [74]. Several approaches based on piezoelectric sensors have been described featuring different materials, geometry, and numbers of required sensing elements to be placed on subjects’ bodies [75,76,77,78,79,80,81,82,83,84,85,86,87,88,89]. Wang et al. described a rigid piezoelectric sensor applied on the wrist to measure PW signals and estimate blood pressure via PW analysis [76]. Obeid et al. used a commercial PWV measurement system, also based on rigid piezoelectric sensors, to estimate blood pressure from radial–digital PWV measurements, obtained by computing the pulse transit time (PTT) between PW signals acquired on the radial artery in the wrist and digital arteries in the fingers [76]. Taranchuk and Pidchenko proposed a particular piezoelectric PW sensor based on a quartz resonator [77]. The sensor is equipped with a funnel that realizes air-coupling between the human body, a membrane electrode and a quartz resonator with a second electrode placed at a certain gap distance. The whole structure is integrated in a Colpitts oscillator circuit, so that when the applied pressure bends the membrane and modulates the gap distance from the electrodes, the frequency of the oscillator circuit varies over time accordingly. Other studies have focused on flexible piezoelectric PW sensors. Kang et al. presented a poly (vinylidene fluoride) (PVDF) piezoelectric sensor with an ad hoc design support structure that closely fits the human wrist to ensure the stable and effective collection of PW signals under continuously varying pressure [78]. The overall device featured a circuit for sensor conditioning, signal acquisition, and real-time processing or Bluetooth data transmission to smartphones or computers. Park et al. designed an earbud-like device featuring a flexible piezoelectric film sensor for in-ear PW signal acquisition and processing, aimed at real-time heart rate monitoring [79]. Dagdeviren et al. proposed a multichannel PW sensor based on an 8 × 8 matrix of lead zirconate titanate (PZT) sensors mounted on a thin, flexible substrate of silicone rubber [80]. Lozano Montero et al. designed a fully printed, biocompatible, ultrathin piezoelectric sensor, made of poly(vinylidene fluoride-trifluoro-ethylene) (PVDF-TrFE), capable of acquiring PW signals from the radial artery in the wrist for accurate blood pressure estimation as compared to a commercial finger-cuff medical BP monitor [81]. Guo et al. presented a high-sensitivity piezoelectric PW sensor with a specific mechanical design, which proved capable of capturing the changes in hemodynamic parameters that occur during premature atrial and/or ventricular contractions, and also for accurate blood pressure monitoring in patients with arrhythmias [82]. Laurila et al. proposed a PVDF-TrFE sensor and charge amplifier, both fully printed on an ultra-thin parylene substrate, for on-skin acquisition of PW signals from radial artery [83]. McLaughlin et al. described the use of two PVDF piezoelectric sensors to simultaneously acquire PW signals from brachial and radial arteries, to eventually obtain brachial–radial PWV measurements [84]. Ghosh and Mandal presented a very interesting design of a bio-assembled piezoelectric sensor made from waste by-product prawn shells, for wearable monitoring of PW signals from the wrist [85]. Bongrain et al. proposed a CMOS-compatible design of a AlN piezoelectric sensor realized on a biocompatible, conformable, extremely thin parylene layer, which proved capable of acquiring PW signals from carotid and radial arteries [86]. Hou et al. described PVDF piezoelectric sensors to simultaneously monitor respiration from the chest and PW signals from the wrist, so as to analyze respiratory-induced variations in PW signals [87]. Polley et al. proposed a piezoelectric sensor cast in a silicone rubber enclosure for simultaneous respiratory and heart rate monitoring from the radial artery, the chest, and the suprasternal notch. The sensor was equipped with Bluetooth communication capabilities and proposed as a wearable sensor for smart triage [88].

Other sensors and techniques have also been proposed to record PWs, which include strain and piezoresistive pressure sensors [89,90,91,92,93,94,95], piezocapacitive pressure sensors [96,97], ferroelectric sensors [98], triboelectric sensors [99], optical force sensors [100,101,102], laser doppler vibrometers [103], accelerometers [3,104], and microwave sensors [105,106].

The performances of PW sensors have been analyzed individually, and, when compared, their results have been found not to be in good agreement, e.g., in terms of the PW morphology and physiological parameters extracted, as reported in [3]. Such a comparison has led to a deeper comprehension of their strengths and weaknesses, and ultimately, to the consideration that a multimodal approach accomplished via sensor fusion would lead to a more robust, reliable, and potentially more informative methodology for PW monitoring. However, apart from various multichannel or multi-site systems proposed in the literature [11,107,108] and the comparison/integration of different sensors applied on different body parts [81,100,109], no true multimodal sensors for PW recording have been proposed yet that acquire PW signals simultaneously from the same body site.

In this study, a true multimodal PW sensor is presented, which was obtained by integrating a piezoelectric forcecardiography (FCG) sensor [110,111,112,113,114,115] and a PPG sensor, thus enabling simultaneous mechanical–optical measurements of PWs from the same site on the body. The novel sensor performance was assessed by measuring finger PWs from five healthy subjects at rest. FCG and PPG sensor performances were compared via both normalized cross-correlation analysis of PW signals and statistical analyses of six well-established morphological parameters of PW. This preliminary study unveiled the existence of a time delay between PW signals acquired by piezoelectric and reflectance-mode optical sensors, which has not previously been described in the literature. It also determined, for the first time, that PW signals acquired simultaneously from the same site via FCG and PPG sensors, as well as their first and second derivatives, share very similar morphologies, as they exhibited very high correlations. Nonetheless, some of their morphological parameters were found not to be in good agreement for all subjects. 

## 2. Materials and Methods

### 2.1. Forcecardiography Sensors

Forcecardiography is a novel technique based on specific wearable force sensors that measure the local forces induced on the chest wall by the mechanical activity of the heart and lungs [110,111,112,113,114,115]. FCG signals were first acquired by means of sensors based on force-sensing resistors (FSR), which have already proved suitable for muscle contraction monitoring [116], gesture recognition [117], and the control of biosignal-based human–machine interfaces [118], such as the “Federica Hand” prosthesis [119,120,121,122] and an upper-limb exoskeleton [123]. The use of such FSR-based sensors has also been demonstrated for continuous respiratory monitoring [114]. Lately, piezoelectric FCG sensors and related conditioning circuits have been presented, and have proved capable of capturing respiration, infrasonic cardiac vibrations, and heart sounds, simultaneously, from a single contact point on the chest [110].

### 2.2. Multimodal PW Sensor

**Figure 1:**
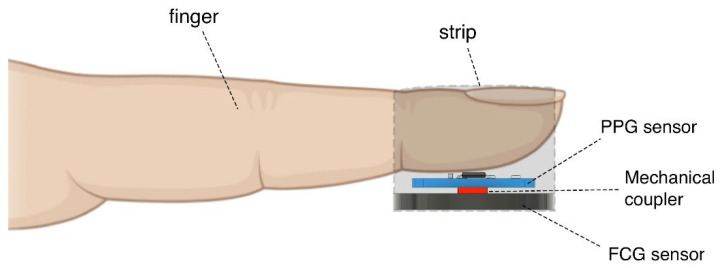
Schematic representation showing the components of the multimodal PW sensor placed on a subject’s finger, namely MAX30102 PPG sensor, piezoelectric FCG sensor, and a flat cylinder acting as a mechanical coupler to ensure reasonable force transduction from the PPG sensor to the FCG sensor.

The multimodal PW sensor was realized by integrating a PPG and a piezoelectric FCG sensor. In particular, the FCG sensor described in [110,112,113] and a PPG sensor board equipped with a MAX30102 chip (Maxim Integrated Products, INC., 160 Rio Robles, San Jose, CA 95134, USA, 408-601-1000) were firmly attached together, in order to make them integral. FCG sensors are usually equipped with dome-shaped mechanical couplers, as they ensure optimal transduction of force from tissues to the sensor. However, in this case, the PPG sensor had to be in direct contact with the finger, so the FCG sensor could not be directly interfaced with the skin via a dome-shaped coupler. To this end, a small, flat cylinder with a diameter of 5 mm was firmly attached both to the back of the PPG sensor board and to the active area of the FCG sensor. In fact, the overall PPG sensor and flat cylinder compound acted as a mechanical coupler for the FCG sensor, thus ensuring a reasonable transduction of force from the finger to the sensor. Figure 1 depicts the structure of the proposed multimodal PW sensor.

The multimodal PW sensor thus realized was applied on subjects’ fingers by means of medical adhesive tape. Since static contact force is known to affect the performance of PW sensors [1,3], and piezoelectric sensors cannot be used for static force measurement, an additional FSR (FSR 402 short, Interlink Electronics, Inc., 1 Jenner Suite 200, Irvine, CA 92618, USA) was attached onto the active area of the piezoelectric FCG sensor, beneath the flat cylinder, to monitor the actual contact force applied by the multimodal PW sensor when mounted on subjects’ fingers. The FSR had an active area with a diameter of 12.7 mm; therefore, a flat cylinder diameter of 5 mm was appropriate. Static calibration of the FSR was performed as in [111,116] to obtain actual force measurements from the FSR sensor readings.

### 2.3. Experimental Measurement Setup and Protocol

Simultaneous recordings of finger PWs and ECG lead-I were obtained, respectively, from the multimodal PW sensor and an ECG board (SparkFun Electronics, Inc., 6333 Dry Creek Parkway, Niwot, CO 80503, USA) based on the AD8232 single-lead heart rate monitor front end (Analog Devices, Inc., 1 Analog Way, Wilmington, MA 01887, USA).

Five healthy subjects (4 males, 1 female; age: 26 ± 2.5 years; height: 177 ± 5.87 cm; weight: 86.0 ± 27.2 kg; BMI: 27.3± 7.60) were enrolled for the experiments. Information on the subjects’ gender, age, height, weight, and BMI are reported in Table 1. The subjects were required to comfortably lie on a medical couch in supine position. The multimodal PW sensor was mounted on the index finger of the right hand with a reasonable contact force, so as to ensure good contact with the finger tissues, without providing excessive stress. Indeed, as reported in the literature [3], if contact force is either too low or too high, the quality of PW signals is substantially impaired. Afterward, ECG electrodes were placed on subjects’ limbs to acquire an ECG lead I. Non-invasive blood pressure (NIBP) measurements were also acquired to ensure that the subjects were not in an altered state. To this end, an NIBP cuff was firmly placed around the left arm and NIBP measurements were acquired via a multiparameter patient monitor (Dynascope DS-7000, Fukuda Denshi, Co., Ltd., 2-35-8 Hongo, Bunkyo-ku, Tokyo, 113-8420, Japan). 

Signals from the multimodal PW sensor and the ECG lead were simultaneously acquired at 200 Hz via an STM32F401RE microcontroller board (STMicroelectronics, Inc., 39 Chemin du Champ des Filles Plan-Les-Ouates, 1228, Switzerland). To this end, the sampling frequency of the MAX30102 digital PPG sensor was set at 200 Hz and the FCG and ECG sensors readings performed by the analog-to-digital converter of the microcontroller (12 bits) were synchronized with the interrupt signal provided by the PPG sensor. This approach did not ensure a perfectly synchronous sampling of all signals (maximum delay lower than 0.5 ms); however, this was not strictly required for this study. An alternative solution to multichannel synchronous sampling is described in [124].

### 2.4. Signal Processing and Analysis

All processing and analyses were carried out in MATLAB^®^ R2017b (MathWorks, Inc., 1 Apple Hill Drive, Natick, MA 01760, USA).

#### 2.4.1. Pre-Processing

PW signals provided by the piezoelectric FCG sensor, the PPG sensor, i.e., red (PPG-R) and infrared (PPG-IR) signals, as well as the ECG lead I, were first resampled at 1 kHz, and then, pre-processed to remove high-frequency noise and baseline oscillations. All PW signals were first low-pass filtered via an 8th-order zero-lag Butterworth filter (4th-order filter applied sequentially in forward and backward directions) with the cut-off frequency set at 20 Hz. Then, a 3rd-order Savitzky–Golay filter [125], with a frame length corresponding to about a 1.5 s interval, was applied to extract baseline oscillations, which were then removed from the signals that had previously been obtained after low-pass filtering. The PW signals provided by the PPG sensor were reversed in amplitude to obtain positive systolic peaks. Finally, the ECG signal was band-pass filtered in the 0.5–40 Hz frequency band via an 8th-order zero-lag Butterworth filter.

#### 2.4.2. Detection of Fiducial Points

**Figure 2:**
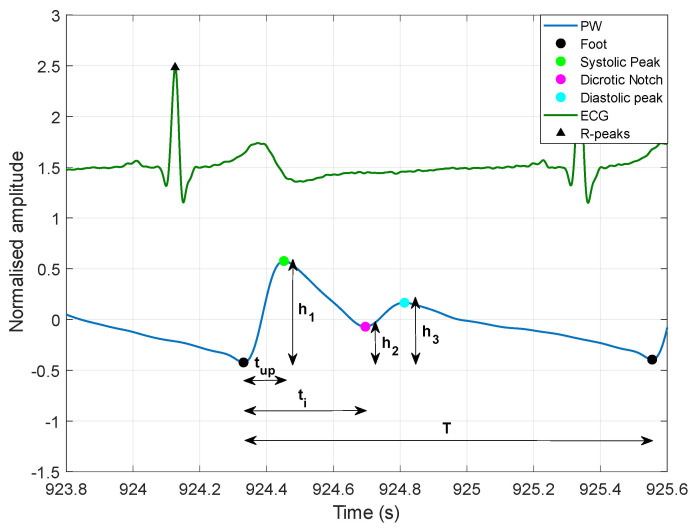
Graphical example of PW fiducial points and morphological parameters.

R-peaks were first located in the ECG signal via the well-known Pan and Thompkins algorithm, implemented in the “BioSigKit” MATLAB^®^ toolbox [126]. Then, the following well-established fiducial points [3] were located in the PW signals provided by the PPG and FCG sensors (see also Figure 2):

- The foot of the systolic rise (referred to as “foot”);
- Systolic peak;
- Dicrotic notch;
- Diastolic peak.

Since the assessment of robustness to motion artifacts was out of the scope of this preliminary study, all signal segments containing motion artifacts in at least one of the PW signals provided by the multimodal PW sensor were excluded from the analyses.

#### 2.4.3. Extraction of PW Morphological Parameters 

After the PW fiducial markers had been located, the following parameters were computed, which are commonly used to characterize the morphology of PW signals [3]:

- ***t_up_***: time distance between the foot and the systolic peak;
- ***t_i_***: time distance between the foot and the dicrotic notch;
- ***T***: time distance between two consecutive feet;
- ***t_up_/T***: ratio of foot time distances from the systolic peak and from subsequent foot;
- ***h_1_***: systolic peak height with respect to the foot;
- ***h_2_***: dicrotic notch height with respect to the foot;
- ***h_3_***: diastolic peak height with respect to the foot;
- ***h_2_/h_1_***: ratio of the dicrotic notch to the systolic peak heights;
- ***h_3_/h_1_***: ratio of the diastolic to systolic peaks heights.

*h_1_*, *h_2_*, and *h_3_* related to PPG and FCG sensors signals were not compared, because they are related to different physical quantities. Instead, the *h_2_/h_1_* and *h_3_/h_1_* ratios are dimensionless quantities that actually characterize the morphology of PW signals, and were considered for the comparison, along with *t_up_*, *t_i_*, *T*, and *t_up_/T*.

#### 2.4.4. Normalized Cross-Correlation

The morphological similarity between the PW signals provided by the PPG and FCG sensors was quantitatively assessed by evaluating their normalized cross-correlation (NCC) [110,111,112,113,114,115]. In particular, NCC was computed both between single corresponding heart beats and between whole signal segments. While the former gives information about beat-by-beat morphological variations in PW signal similarity regardless of amplitude variations, the latter also takes into account the amplitude modulations that are usually observed over time in PW signals [1,2,3].

Single heart beats were segmented in each PW signal by considering the time intervals between two consecutive feet. Then, the normalized cross-correlation function (NCCF) of PW signals provided by FCG and PPG sensors was computed between segments corresponding to the same heart beats. Afterwards, the NCC was obtained as the maximum of the NCCF. For whole signal segments, the NCCF was first computed; then, the maximum of the NCCF was located. Its value corresponded to the NCC, while its position gave information on the average time lag between the analyzed signals.

#### 2.4.5. Statistical Analyses 

Regression, correlation and Bland–Altman analyses were carried out via the MATLAB^®^ function “*bland-altman-and-correlation-plot*” [127] to compare the morphological parameters extracted from PW signals acquired by the FCG sensor and the PPG sensor. These statistical analyses were performed either on the dataset of parameters acquired from each single subject or on a combined dataset obtained by joining the parameters of all subjects. 

## 3. Results

### 3.1. Time Delays between Fiducial Markers

**Figure 3:**
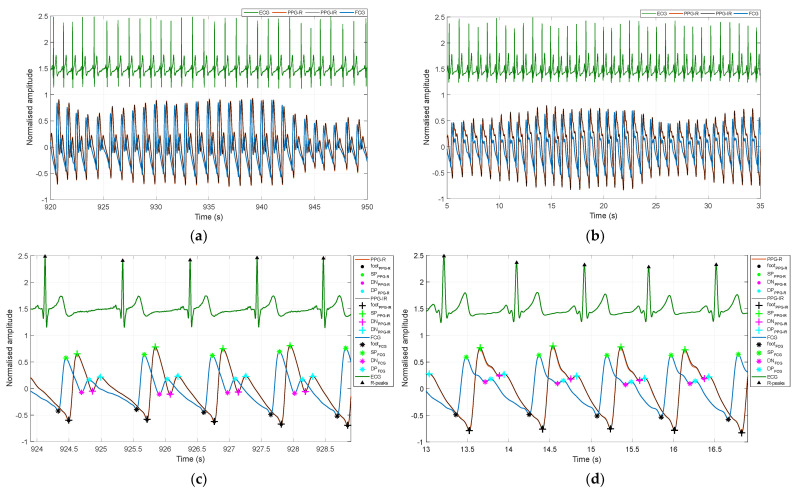
Example of PW signals acquired by the PPG sensor (red and infrared) and the piezoelectric FCG sensor, along with the concurrently acquired ECG lead I. ECG R-peaks and the four fiducial markers located in PW signals are also depicted. A delay between the PPG and FCG sensor signals can be clearly observed. (**a**) Signals acquired in subject #1; (**b**) signals acquired in subject #3; (**c**) detail on four heart beats from the signal shown in panel (**a**) with the localized fiducial markers; (**d**) detail on four heart beats from the signal shown in panel (**b**) with the localized fiducial markers.

Figure 3 shows an example of ECG and PW signals acquired from subjects #1 and #3. Figure 3a,b show 30-s segments where it can be clearly observed that piezoelectric PWs (blue line) and optical PWs (red and black lines) are very similar, the latter being consistently delayed with respect to the former. A detail on PW morphology is depicted in Figure 3c,d, which show 5-second PW signal segments, along with localized fiducial markers. A delay between corresponding markers can also be observed.

The pulse arrival time (PAT), considered as the time interval between PW systolic peaks and ECG R-peaks, across all subjects was 483 ± 20.2 ms for PPG-R (red light), 485 ± 19.4 ms for PPG-IR (infrared light), and 315 ± 22.0 ms for the piezoelectric FCG sensor. The average PATs of the PPG signals were found to be in agreement with those reported in [128], while the PATs of the piezoelectric PW signals turned out to be consistently lower, which further highlights the existence of a time delay between optical and piezoelectric PW signals. This delay was estimated by considering the time intervals between corresponding fiducial markers of piezoelectric and PPG signals, which are outlined in Table 2.

**Figure 4:**
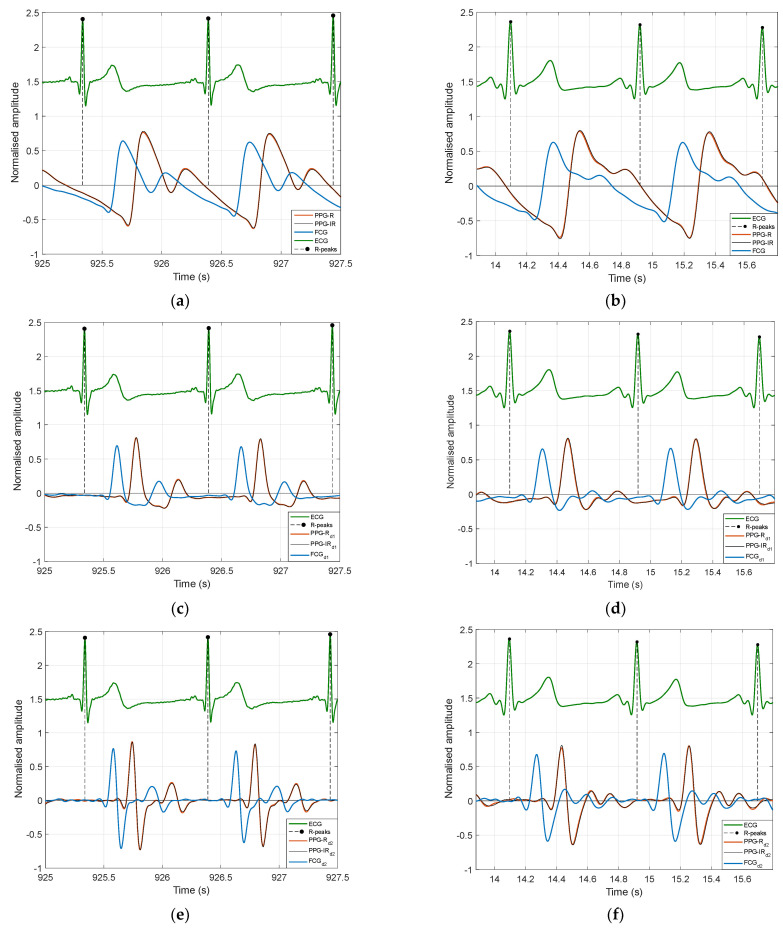
Comparison of PW signals and related first and second derivatives, acquired by the PPG sensor (red and infrared photodetectors) and the piezoelectric FCG sensor, along with the concurrently acquired ECG lead I. Signals acquired in subjects #1 and #3 are depicted in the first and second columns, respectively. The original PW signal, first derivative, and second derivative of both subjects are depicted in the first (**a**,**b**), second (**c**,**d**), and third (**e**,**f**) rows, respectively.

Figure 4 shows 5-s segments of the original PW signals acquired from subjects #1 and #3, along with the first and second derivatives.

### 3.2. Normalized Cross-Correlation

The NCC between piezoelectric PW signals and optical PW signals was first computed for each single heartbeat. The means and SDs of the computed beat-by-beat NCC are reported in Table 3. Table 4 instead reports the NCC computed between the whole piezoelectric and optical PW signals, along with the related time lags. As expected, the NCC time lags were in very good agreement with those computed between corresponding PW fiducial markers (see Table 2).

### 3.3. Morphological Parameters of PW

The means and SDs of the morphological parameters extracted from the optical and piezoelectric PW signals are reported, for each subject, in Table 5. Table 6 outlines, for each subject, the results of the regression, correlation, and Bland–Altman analyses that were carried out to compare the morphological parameters extracted from piezoelectric PW signals against those extracted from optical PW signals. Table 7 shows the results achieved by performing the same statistical analyses on the combined-parameters dataset (obtained by joining the data of all subjects).

## 4. Discussion

To the best of our knowledge, this study addressed, for the first time in the literature, the simultaneous measurement of finger-pulse waveforms via a true multimodal sensor, realized by integrating a reflectance-mode PPG and a piezoelectric FCG sensor, which acquire PW signals from the same site. The preliminary results of this study unveiled the existence of a time delay between the PW signals recorded by a reflectance-mode optical sensor and a piezoelectric sensor, which has also not been previously described in the literature. Therefore, these preliminary results suggest that the changes in blood vessels’ lumens and in the optical reflectance of tissues, due to local changes in blood pressure in the finger, have different time dynamics. However, the actual mechanisms behind this phenomenon are still unclear, and undoubtedly deserve deeper investigation, which was outside of the scope of this study.

The results also showed, for the first time, that PW signals acquired by a PPG sensor and a piezoelectric FCG sensor from the same site share very similar morphology, as they had very high normalized cross-correlation scores. Indeed, the beat-by-beat NCCs turned out to be 0.990 ± 0.005, and always in excess of 0.98 for all subjects, thus indicating very high and stable morphological similarity between PWs provided by FCG and PPG sensors. The NCCs computed between whole signals turned out to be 0.984 ± 0.007, and always in excess of 0.98, except for subject #4 (NCC > 0.97), thus showing that the amplitude modulations exhibited by PW signals acquired via different sensors were also very similar.

Reasonable agreement was generally found between the PW parameters extracted from FCG and PPG sensors signals, apart from the time interval between the feet and the systolic peaks (*t_up_* parameter), for which the greatest disagreement was observed. In particular, a statistically significant bias (*p* < 0.0001) was found for the diastolic-to-systolic peak ratio (*h_3_/h_1_*) and the dicrotic notch-to-systolic peak ratio (*h_2_/h_1_*), which turned out to be consistently lower in PW signals acquired by the piezoelectric FCG sensor with respect to signals acquired by the PPG sensor. This suggests that the relative height of the systolic peak was higher in piezoelectric PW signals, which could also be qualitatively assessed via visual inspection of the signals in Figure 3.

As a preliminary investigation, this study has some limitations, which will be the subject of future studies. Only a small cohort of healthy volunteers was considered for this study, and its preliminary results need to be confirmed on a larger cohort, also including actual patients; this would enable us to verify if hemodynamic changes, both physiological and pathological, produce the same effects on the morphology of PW signals acquired by PPG and piezoelectric FCG sensors. In addition, only PW acquired from the finger were analyzed. The performance assessment of the proposed multimodal PW sensor could also be extended to PWs acquired from radial, brachial, carotid, iliac, and femoral arteries. The unexpected delay observed between the PW signals acquired by PPG and FCG sensors must be further investigated by using different PPG sensors (reflective and transmissive types) and identifying potential physical mechanisms that can explain the experimental data. The acquisition of PW signals was limited to subjects at rest, so as to analyze the multimodal PW sensor performances in the best experimental conditions. Sensor performances should also be assessed in subjects undergoing stress testing, such as the cold pressor test [129], mental arithmetic test [130], Valsalva maneuver [131], and various physical exercises, which are commonly adopted to elicit various hemodynamic changes. Finally, extending the analysis to subjects performing various physical exercises is also important to assess the robustness to motion artifacts of PPG and piezoelectric FCG sensors integrated in the proposed multimodal PW sensor. All these analyses could also lead to deeper comprehension of the strengths and weaknesses of these sensors and highlight opportunities for the development of sensor fusion strategies; these would be aimed at exploiting the multimodal nature of the proposed sensor to overcome the current limitations of existing PW sensing technologies and achieve superior accuracy, reliability, and reproducibility.

## Figures and Tables

**Table 1:** Subjects’ demographics.

Subject	Gender	Age (Years)	Height (cm)	Weight (kg)	BMI
1	Male	23	181	75	22.89
2	Female	27	175	63	20.57
3	Male	30	168	82	29.05
4	Male	26	183	133	39.71
5	Male	26	178	77	24.30

**Table 2:** Mean and SD of time delays (in milliseconds) between PW fiducial markers of FCG and PPG.

Subject	Foot		Systolic Peak		Dicrotic Notch		Diastolic Peak	
	PPG-R	PPG-IR	PPG-R	PPG-IR	PPG-R	PPG-IR	PPG-R	PPG-IR
1	163 ± 1.12	165 ± 1.15	168 ± 4.39	170 ± 4.77	166 ± 1.52	168 ± 1.33	164 ± 3.66	166 ± 3.94
2	162 ± 2.58	164 ± 2.18	162 ± 8.77	166 ± 11.3	167 ± 6.27	171 ± 6.36	167 ± 6.27	171 ± 6.36
3	163 ± 4.44	165 ± 4.53	174 ± 5.38	177 ± 6.17	178 ± 26.3	183 ± 26.3	166 ± 8.77	168 ± 8.61
4	165 ± 11.3	166 ± 15.7	170 ± 5.92	171 ± 5.72	169 ± 3.20	171 ± 2.91	167 ± 4.30	170 ± 4.03
5	164 ± 2.35	165 ± 2.42	165 ± 6.67	165 ± 5.79	163 ± 4.94	166 ± 4.99	162 ± 6.30	163 ± 6.47

**Table 3:** Mean and SD of beat-by-beat normalized cross-correlations between PW signals acquired by FCG and PPG-R sensors.

Subject	Original PW	First Derivative of PW	Second Derivative of PW
1	0.991 ± 0.004	0.995 ± 0.003	0.995 ± 0.005
2	0.996 ± 0.005	0.994 ± 0.004	0.990 ± 0.007
3	0.983 ± 0.009	0.987 ± 0.007	0.991 ± 0.004
4	0.991 ± 0.007	0.988 ± 0.009	0.981 ± 0.012
5	0.989 ± 0.008	0.990 ± 0.007	0.984 ± 0.011

**Table 4:** Normalized cross-correlations and time lags between whole PW signals acquired by FCG and PPG-R sensors.

Subject	Original PW		First Derivative of PW		Second Derivative of PW	
	NCC	Lag (ms)	NCC	Lag (ms)	NCC	Lag (ms)
1	0.991	167	0.991	163	0.991	161
2	0.990	163	0.990	161	0.986	161
3	0.981	168	0.981	162	0.984	160
4	0.975	165	0.974	165	0.970	166
5	0.981	165	0.980	161	0.975	160

**Table 5:** Morphological parameters extracted from PW signals acquired by FCG and PPG sensors.

Subject		*t_up_ (ms)*	*t_i_ (ms)*	*T (ms)*	*t_up_/T*	*h_2_/h_1_*	*h_3_/h_1_*
1	**PPG-R**	136 ± 4.88	366 ± 7.47	965 ± 106	0.143 ± 0.0167	0.393 ± 0.0594	0.600 ± 0.0482
	**PPG-IR**	137 ± 5.11	367 ± 7.46	965 ± 106	0.143 ± 0.0168	0.390 ± 0.0577	0.589 ± 0.0447
	**PIEZO**	131 ± 6.18	363 ± 7.48	965 ± 106	0.138 ± 0.0172	0.302 ± 0.0545	0.525 ± 0.0461
2	**PPG-R**	122 ± 6.66	374 ± 7.35	995 ± 121	0.125 ± 0.0165	0.631 ± 0.0583	0.739 ± 0.0733
	**PPG-IR**	125 ± 9.05	376 ± 7.59	995 ± 122	0.128 ± 0.0175	0.650 ± 0.0592	0.751 ± 0.0743
	**PIEZO**	123 ± 8.30	373 ± 9.09	995 ± 121	0.126 ± 0.0175	0.593 ± 0.0684	0.694 ± 0.0787
3	**PPG-R**	127 ± 3.34	359 ± 8.45	894 ± 70.8	0.143 ± 0.0099	0.671 ± 0.0515	0.699 ± 0.0545
	**PPG-IR**	129 ± 3.26	362 ± 8.17	894 ± 70.8	0.145 ± 0.0100	0.666 ± 0.0486	0.688 ± 0.0509
	**PIEZO**	119 ± 3.79	353 ± 9.17	894 ± 71.0	0.134 ± 0.0109	0.546 ± 0.0412	0.589 ± 0.0426
4	**PPG-R**	121 ± 8.54	339 ± 11.4	863 ± 81.3	0.142 ± 0.0159	0.190 ± 0.0787	0.490 ± 0.110
	**PPG-IR**	121 ± 7.96	340 ± 11.1	863 ± 81.2	0.142 ± 0.0158	0.185 ± 0.0754	0.488 ± 0.104
	**PIEZO**	125 ± 10.9	335 ± 12.5	863 ± 80.7	0.146 ± 0.0198	0.137 ± 0.0809	0.505 ± 0.0549
5	**PPG-R**	131 ± 6.60	334 ± 8.09	903 ± 92.9	0.147 ± 0.0165	0.531 ± 0.0842	0.685 ± 0.0530
	**PPG-IR**	130 ± 5.28	335 ± 7.94	903 ± 93.0	0.145 ± 0.0156	0.510 ± 0.0762	0.659 ± 0.0502
	**PIEZO**	130 ± 4.39	334 ± 8.35	903 ± 92.8	0.145 ± 0.0153	0.434 ± 0.0659	0.583 ± 0.0472

**Table 6:** Results of the regression, correlation, and Bland–Altman analyses that were carried out to compare the morphological parameters of PW signals provided by FCG and PPG sensors for each subject. Intercept, bias, and LoAs for *t_up_*, *t_i_*, and *T* are expressed in milliseconds. Non-significant bias is indicated as “NS”.

Subject	Parameter	*t_up_*		*t_i_*		*T*		*t_up_/T*		*h_2_/h_1_*		*h_3_/h_1_*	
		R	IR	R	IR	R	IR	R	IR	R	IR	R	IR
1	Slope	0.910	0.820	0.975	0.980	1.000	1.000	0.996	0.980	0.868	0.879	0.905	0.945
	Intercept	7.13	19.2	6.21	3.7	−0.377	−0.632	−0.005	−0.003	−0.039	−0.041	−0.018	−0.031
	R^2^	0.516	0.46	0.949	0.955	1.000	1.000	0.928	0.916	0.893	0.866	0.894	0.839
	Bias	−5.18	−5.39	−2.90	−3.63	NS	NS	−0.005	−0.006	−0.091	−0.088	−0.075	−0.064
	*p*-value	^c^	^c^	^c^	^c^	0.885	0.890	^c^	^c^	^c^	^c^	^c^	^c^
	LoAs	±8.47	±9.08	±3.34	±3.13	±2.92	±3.05	±0.009	±0.01	±0.04	±0.04	±0.03	±0.04
2	Slope	0.260	0.0765	1.01	0.966	0.993	0.995	0.886	0.769	0.913	0.869	0.751	0.739
	Intercept	91.3	113	−4.78	10.4	6.80	4.90	0.015	0.027	0.017	0.029	0.139	0.139
	R^2^	0.044	0.007	0.671	0.651	1.000	1.000	0.695	0.590	0.606	0.564	0.489	0.487
	Bias	0.774	−2.24	−0.415	−2.45	NS	NS	0.001	−0.002	−0.037	−0.056	−0.045	−0.057
	*p*-value	^a^	^c^	^a^	^c^	0.955	0.964	^a^	^c^	^c^	^c^	^c^	^c^
	LoAs	18.6	23.1	10.2	10.5	4.44	4.19	0.019	0.023	0.085	0.090	0.116	0.117
3	Slope	0.632	0.501	0.956	0.982	1.00	1.00	1.04	1.01	0.644	0.689	0.689	0.738
	Intercept	38.6	54.2	9.88	−2.67	−2.3	−2.51	−0.015	−0.013	0.114	0.088	0.108	0.082
	R^2^	0.31	0.186	0.778	0.767	1.000	1.000	0.897	0.867	0.65	0.661	0.78	0.779
	Bias	−8.25	−10.1	−5.86	−9.15	NS	NS	−0.009	−0.011	−0.125	−0.12	−0.109	−0.099
	*p*-value	^c^	^c^	^c^	^c^	0.957	0.953	^c^	^c^	^c^	^c^	^c^	^c^
	LoAs	6.62	7.42	8.5	8.68	2.62	2.44	0.007	0.008	0.060	0.056	0.051	0.047
4	Slope	0.98	1.1	1.02	1.07	0.993	0.993	1.15	1.17	0.949	1	0.465	0.493
	Intercept	5.9	−7.94	−12.6	−27.2	6.25	5.89	−0.017	−0.020	−0.043	−0.048	0.277	0.264
	R^2^	0.589	0.639	0.873	0.896	0.999	0.999	0.852	0.877	0.852	0.872	0.877	0.877
	Bias	3.48	3.61	−4.38	−4.95	0.0523	0.0392	0.004	0.005	−0.053	−0.048	0.014	0.016
	*p*-value	^c^	^c^	^c^	^c^	0.808	0.860	^c^	^c^	^c^	^c^	^c^	^c^
	LoAs	13.7	12.9	8.75	8.05	5.22	5.38	0.016	0.015	0.062	0.057	0.122	0.110
5	Slope	0.248	0.302	0.835	0.845	0.998	0.997	0.836	0.905	0.74	0.815	0.794	0.822
	Intercept	97.3	90.7	55.5	51.3	1.93	2.53	0.0225	0.0142	0.0411	0.0186	0.0387	0.041
	R^2^	0.14	0.132	0.655	0.646	0.999	0.999	0.812	0.847	0.893	0.887	0.794	0.766
	Bias	−1.28	NS	NS	−0.768	NS	NS	−0.001	0.0004	−0.097	−0.076	−0.103	−0.076
	*p*-value	^a^	0.247	0.190	^a^	0.940	0.956	^b^	0.278	^c^	^c^	^c^	^c^
	LoAs	12.6	10.8	9.96	10	6.26	6.45	0.014	0.012	0.060	0.051	0.047	0.048

^a^*p* < 0.05; ^b^ *p* < 0.001; ^c^ *p* < 0.0001

**Table 7:** Results of the regression, correlation, and Bland–Altman analyses that were carried out to compare the morphological parameters of PW signals provided by FCG and PPG sensors for all subjects. Intercept, bias, and LoAs for *t_up_*, *t_i_*, and *T* are expressed in milliseconds. Non-significant bias is indicated as “NS”.

Parameter	*t_up_*		*t_i_*		*T*		*t_up_/T*		*h_2_/h_1_*		*h_3_/h_1_*	
	R	IR	R	IR	R	IR	R	IR	R	IR	R	IR
Slope	0.472	0.304	1.010	0.989	0.996	0.997	0.887	0.868	0.999	0.969	0.788	0.784
Intercept	65.5	86.1	−5.78	0.762	4.10	3.06	0.014	0.015	−0.062	−0.051	0.087	0.090
R^2^	0.210	0.098	0.921	0.915	1.000	1.000	0.775	0.711	0.905	0.927	0.680	0.736
Bias	−0.915	−2.52	−1.48	−3.24	NS	NS	−0.001	−0.003	−0.063	−0.069	−0.059	−0.059
*p*-value	^c^	^c^	^c^	^c^	0.919	0.934	^c^	^c^	^c^	^c^	^c^	^c^
LoAs	±16.8	±19.5	±10.2	±10.5	±4.55	±4.48	±0.018	±0.020	±0.096	±0.085	±0.117	±0.109

^a^*p* < 0.05; ^b^ *p* < 0.001; ^c^ *p* < 0.0001

## Data Availability

The datasets presented in this article are not readily available because informed consent from the subjects involved was obtained only for this study and not for public availability. Requests to access the datasets should be directed to E.A. (emilio.andreozzi@unina.it) or M.R. (michele.riccio@unina.it).
